# Molecular Regulation of Secondary Hair Follicle Stem Cell by S100a4 in Cashmere Goat

**DOI:** 10.3390/ijms27020849

**Published:** 2026-01-15

**Authors:** Xinyue Liang, Bohan Liu, Jiayi Wang, Yanlei Liu, Yiping Wei, Hongji Yu, Junpeng Zhang, Shuyi Zhang, Huiling Xue

**Affiliations:** College of Animal Science and Veterinary Medicine, Shenyang Agricultural University (SYAU), Shenyang 110866, China

**Keywords:** S100a4, cashmere, hair follicle stem cell, keratin

## Abstract

Secondary hair follicle stem cells (HFSCs) are essential for cashmere fiber regeneration, yet the molecular mechanisms governing their activation and lineage progression remain poorly understood. Here, we identify S100a4 as a key regulator of secondary HFSCs in cashmere goat. *S100a4* expression peaks during anagen and is markedly enriched in secondary HFSCs relative to hair matrix cells (HMCs), suggesting a role in initiating follicle regeneration. Functional assays show that *S100a4* promotes HFSCs into a dynamically regulated state that activates stem cell competence while facilitating differentiation, with overexpression upregulating epidermal and follicular differentiation markers (*Ivl*, *Cux1*, *K14*, *Klk5*), as well as pluripotency genes (*Itga6*, *Krt15*), while knockdown suppresses these programs. Proteomic analysis further reveals direct interactions between S100A4 and keratins critical for hair follicle and epidermal development (KRT5, KRT14, KRT8, KRT18), suggesting a structural and regulatory interface through which S100A4 modulates HFSC fate. Collectively, these results establish S100a4 as a central modulator of secondary HFSC function and provide mechanistic insight into the molecular control of hair follicle regeneration, with potential implications for improving cashmere fiber production.

## 1. Introduction

Cashmere is a fine, resilient fiber of high economic value, produced by secondary hair follicles (SHFs) [1,2], which undergo annual cycles of regeneration driven by secondary hair follicle stem cells (HFSCs). Follicle regeneration depends on the activation, proliferation, and differentiation of these stem cells. HFSCs are capable of self-renewal and multipotent differentiation potential, enabling them to sustain the hair cycle and shape follicle growth. They can migrate to the hair bulb and differentiate into hair matrix cells (HMCs), or move toward the upper follicle to support epidermal renewal and sebaceous gland maintenance. The morphogenesis and cyclic renewal of hair follicles further rely on coordinated regulators and orchestrated signaling pathways, which activates HFSCs and promotes follicle growth.

S100a4 is a member of the S100 family of calcium-binding proteins and can function both intracellularly and extracellularly [3,4]. Intracellularly, it forms non-covalent homodimers, while extracellular S100A4 is secreted as a covalently linked dimer with potent regulatory activity capable of influencing gene expression [5,6]. S100A4 has been implicated in cancer progression and metastasis [7], as well as tissue fibrosis and inflammatory disorders [8]. It is highly expressed in many tumor types and contributes to proliferation, migration, and survival [9,10,11]. Within the skin, S100A4 is detected in the follicular bulge and hair germ [12], and its dysregulation has been associated with systemic sclerosis-related skin fibrosis and psoriasis [13,14]. However, its role in hair follicle development and HFSC function—particularly in cashmere-producing follicles—remains largely unknown.

Keratins are the major intermediate filament proteins of epithelial tissues [15]. They assemble as obligate heteropolymers, with specific keratin pairs providing structural stability in specific epithelial compartments. KRT5/KRT14 characterize basal keratinocytes [16], while KRT1/KRT10 mark differentiating epidermis [17]. These filament networks are essential for mechanical resilience, stress protection, and the regulation of signaling pathways involved in wound repair and apoptosis [18]. In the hair follicle, keratins form the core structural framework of wool and cashmere fibers. In addition to their structural functions, keratins contribute to cell fate decisions and the regulation of signaling. KRT5/KRT14 and KRT8/KRT18 serve as core structural components of epithelial cells and markers of lineage commitment. During epidermal stratification and hair follicle development, cells transition from KRT8/KRT18 to KRT5/KRT14 expression as they commit to specific lineages. These keratins also contribute to pathways essential for tissue regeneration. Both KRT5/KRT14 and KRT8/KRT18 interact with components of the Wnt/β-catenin pathway, underscoring their broader regulatory roles in epithelial homeostasis and morphogenesis [19,20,21].

S100 family proteins act as multifunctional regulators that translate calcium signals into cytoskeletal remodeling and cell fate determination. S100A4 interacts with actin, non-muscle myosin IIA and tropomyosin, modulating filament assembly, turnover, and cytoskeletal organization. These interactions confer cytoskeletal plasticity, enabling dynamic restructuring of the cell’s architecture during migration, wound repair, and differentiation [6,22]. These interactions confer cytoskeletal plasticity, enabling dynamic restructuring of the cell’s architecture during migration, wound repair, and differentiation. Mechanistically, S100A4 modulates myosin II-dependent contractility and actin filament dynamics, influencing tension-sensitive pathways such as RhoA, MAPK/ERK, and NF-κB that regulate gene expression linked to lineage specification [23,24,25]. Similarly, the S100A8/A9 heterodimer interacts with keratin 8, contributing to the reorganization of keratin networks during differentiation and dermatological inflammatory activation [26]. These interactions fine-tune cytoskeletal integrity and signaling, creating a feedback loop that integrates mechanical cues with transcriptional programs governing differentiation. Thus, through their ability to couple calcium sensing with cytoskeletal remodeling, S100A4 and S100A8/A9 function as molecular rheostats that coordinate cytoskeletal dynamics and differentiation across multiple tissue contexts.

In this study, we identify S100a4 as a key modulator of secondary HFSC activity. We demonstrate that S100a4 promotes HFSC differentiation while modulating stem cell pluripotency, yet exerts no detectable effect on proliferation. Mechanistically, S100A4 interacts directly with keratin proteins to influence HFSC behavior. These findings reveal a previously unrecognized role of S100a4 in secondary HFSC and suggest its potential as a target for promoting cashmere growth.

## 2. Results

### 2.1. Isolation and Culture of Secondary Hair Follicle Stem Cells from Cashmere Goats

To investigate the regulatory mechanisms governing secondary HFSC behavior, we first isolated secondary HFSCs from cashmere goat skin and established a stable in vitro culture system. The isolation procedure yielded a morphologically homogeneous population of small, tightly adherent cells with a high nucleus-to-cytoplasm ratio, consistent with previously described characteristics of HFSCs.

**Figure 1:**
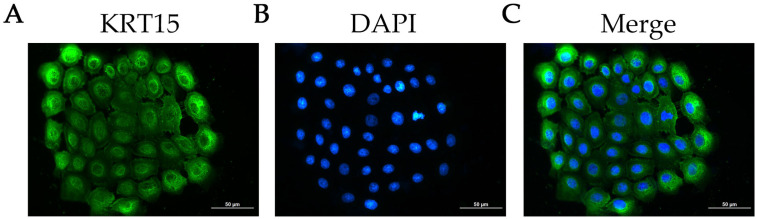
Identification of cultured SHFSCs in vitro. (**A**) Immunofluorescence staining for KRT15 (green). (**B**) Nuclear staining with DAPI (blue). (**C**) Merged image. Scale bar: 50 μm.

To verify the identity and purity of the isolated cells, we examined the expression of KRT15, a well-established HFSC marker. Immunofluorescence staining showed strong KRT15 positivity in the majority of cells (Figure 1), confirming the successful enrichment of secondary HFSCs (>98). The reliable acquisition of a high-purity HFSC population provided a robust foundation for subsequent functional assays investigating S100a4.

### 2.2. S100a4 Is Highly Expressed During Anagen and Enriched in Secondary HFSCs

**Figure 2:**
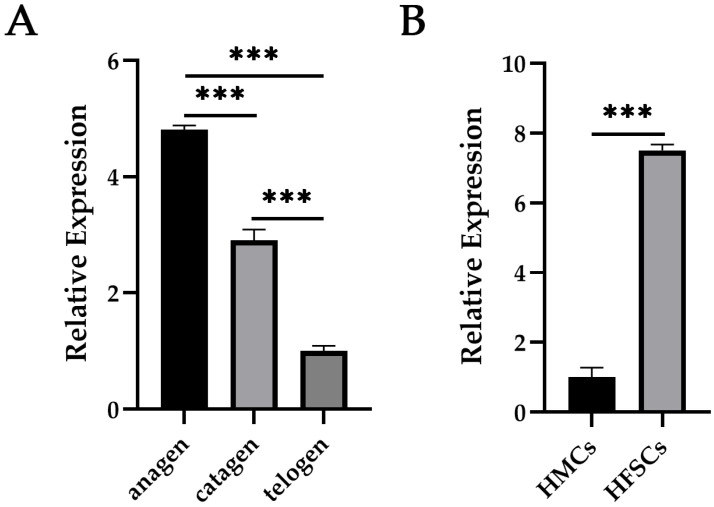
Temporal–spatial expression of *S100a4* in SHFs of cashmere goats. (**A**) Dynamic expression of *S100a4* in SHFs across anagen, catagen, and telogen phase. (**B**) *S100a4* expression in secondary HFSCs and HMCs, respectively. *n* = 3 biological replicates for all groups. Data are presented as means ± SD in all bar graphs. *** *p* < 0.001. Statistical significance was determined by two-sided Student’s *t*-test.

To characterize the potential involvement of *S100a4* in hair follicle cycling, we assessed its expression in secondary hair follicles across three distinct phases—anagen (September), catagen (December), and telogen (February). Quantitative analysis revealed a progressively decline in *S100a4* expression from anagen to telogen, with statistically significant differences between each phase (Figure 2A). The markedly elevated expression during anagen suggests that S100a4 may contribute to the initiation or maintenance of active follicle regeneration.

We next compared *S100a4* expression between two follicular cell populations—secondary HFSCs and hair matrix cells (HMCs). *S100a4* levels were substantially higher in HFSCs (Figure 2B), indicating a stem cell enrichment pattern. This differential expression supports the hypothesis that S100a4 plays a regulatory role in HFSC activation, early lineage specification, or cellular transitions related to follicle cycling.

### 2.3. S100a4 Activates Secondary HFSCs and Facilitates Differentiation

**Figure 3:**
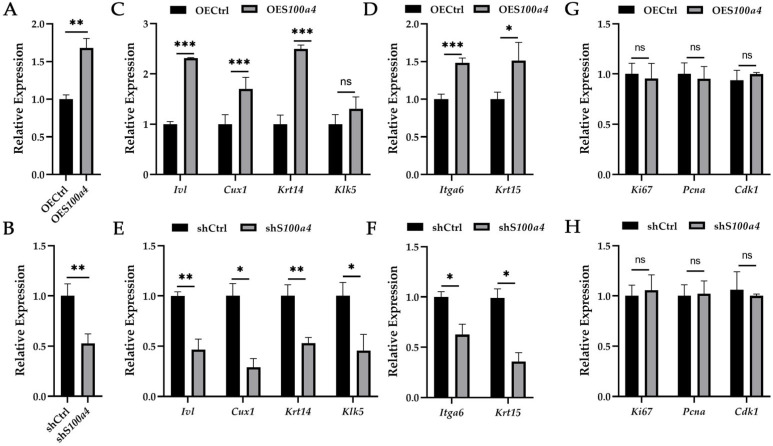
S100a4 regulate the activity of secondary HFSCs. (**A**,**B**) Expression of *S100a4* after *S100a4* overexpression (**A**) or knockdown (**B**). (**C**,**D**) Relative expression of differentiation (**C**) and pluripotency-related genes (**D**) as indicated *S100a4* over expression. (**E**,**F**) Relative expression of differentiation (**E**) and pluripotency-related genes (**F**) as indicated following *S100a4* knockdown. (**G**,**H**) Relative expression of proliferation markers (*Ki67*, *Pcna*, *Cdk1*) after *S100a4* over expression (**G**) or *S100a4* knockdown (**H**). *n* = 3 biological replicates for all groups. Data are presented as means ± SD in all bar graphs. * *p* < 0.05, ** *p* < 0.01, *** *p* < 0.001. Statistical significance was determined by two-sided Student’s *t*-test. OECtrl: overexpression control; OE*S100a4*: *S100a4* overexpression; shCtrl: knockdown control; sh*S100a4*: *S100a4* knockdown.

To determine the functional role of S100a4 in secondary HFSCs, we modulated its expression in vitro using both gain- and loss-of-function approaches. Following *S100a4* overexpression, secondary HFSCs displayed significantly elevated levels of epidermal and follicular differentiation markers (*Ivl*, *Cux1*, *Krt14*, *Klk5*) (Figure 3C). Concurrently, pluripotency-associated markers (*Itga6*, *Krt15*) were also upregulated (Figure 3D), suggesting that S100a4 promotes HFSCs into a dynamically regulated state that activates stem cell competence while facilitating differentiation.

In contrast, *S100a4* knockdown led to marked decreases in both differentiation-related and pluripotency-related genes (Figure 3E,F), indicating impaired lineage commitment along with diminished stem cell characteristics. These findings suggest that S100a4 may function as a molecular rheostat that activates HFSCs in a competent, multipotent state while facilitating their appropriate differentiation during follicle regeneration.

Unlike its clear effects on differentiation and pluripotency, S100a4 did not significantly influence HFSC proliferation. Neither overexpression nor silencing of *S100a4* altered the expression of key proliferation markers, including *Ki67*, *Pcna*, and *Cdk1* (Figure 3G,H).

Collectively, these results identify S100a4 as a multifunctional regulator of secondary HFSCs that regulate stem cell activation and differentiation but exerts no detectable effect on proliferation—properties that are fundamental for orchestrating efficient hair follicle regeneration.

### 2.4. S100A4 Modulates and Interacts with Keratins Involved in Hair Follicle and Epidermal Development

**Figure 4:**
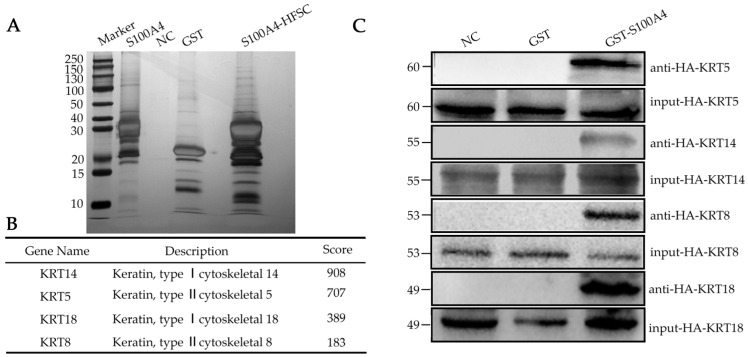
S100A4 interacts with Keratins in secondary HFSCs. (**A**) S100A4 GST pull down in secondary HFSCs. (**B**) S100A4 interact with keratins. (**C**) Immunoblot analysis showing pull-down of keratins KRT5, KRT14, KRT8, and KRT18 by S100A4 in secondary HFSCs.

To elucidate the mechanistic basis of S100A4 function, we performed GST pull-down assays combined with LC–MS/MS to identify S100A4-interacting proteins in secondary HFSCs (Figure 4A). Proteomic analysis revealed that several keratin family members—including KRT5, KRT14, KRT8, and KRT18—were specifically enriched in S100A4-bound fractions (Figure 4B). These keratins are core structural components of epithelial intermediate filaments and serve as markers of lineage commitment during epidermal and hair follicle development.

Western blot validation using eukaryotically expressed keratin proteins confirmed direct physical interactions between S100A4 and each of these keratins (Figure 4C). This finding provides mechanistic insight into S100A4 function, suggesting that S100A4 may regulate HFSC fate by modulating keratin dynamics, cytoskeletal organization, or keratin-mediated signaling pathways.

Given that KRT5/KRT14 mark basal HFSC-like epithelial populations and KRT8/KRT18 are associated with early epithelial progenitors, the interaction network uncovered here implies that S100A4 may influence transitions between distinct HFSC states. By engaging with multiple keratin partners, S100A4 may coordinate structural and signaling events that guide HFSCs toward appropriate epidermal or follicular lineages.

## 3. Discussion

Our findings identify S100a4 as a previously unrecognized regulator of secondary HFSC activity. *S100a4* is highly enriched in secondary HFSCs and exhibits its strongest expression during the anagen phase of secondary hair follicles, consistent with a role in initiating and sustaining the regenerative cycle. Functionally, S100A4 upregulation during early anagen reflects a transient activation state in which HFSCs exhibit increased plasticity, maintaining stem cell competence while permitting differentiation.

These results contrast with extensive evidence from other biological systems in which S100a4 modulates the cell cycle, proliferation, apoptosis, and migration across diverse cell types [27,28]. Likewise, they differ from its well-established role in tumor progression, where elevated S100a4 expression is frequently associated with enhanced proliferative capacity and increased metastatic potential [29,30,31]. In tumor progression, S100A4 interacts with key signaling mediators such as EGFR and β-catenin [31,32], which are critically involved in keratinocyte differentiation and follicular proliferation. The divergence between our findings and previous reports suggests that S100a4 may exert context-dependent, tissue-specific functions, acting not as a general proliferative driver but as a fine-tuned regulator of stem cell state and lineage commitment within the hair follicle niche.

S100A4 appears to play a conserved regulatory role in hair follicle cycling across multiple mammalian species. During the mouse hair cycle, S100a4 exhibits a distinct spatiotemporal expression pattern that correlates with HFSC fate. As follicles transition from telogen to catagen, S100a4 localizes specifically to the bulge region and remains detectable throughout catagen. At the onset of anagen, *S100a4* mRNA is re-expressed in the bulge during both spontaneous regeneration and damage-induced activation, such as plucking injury [33]. In human, *S100a4* also preferentially expressed in bulge cells compared to differentiated HF keratinocytes (KC) [34]. In cashmere goats, S100A4 expression has been observed in the secondary hair follicles, especially during the new hair initiation, implying a conserved role in follicular cycling among mammals [35]. These dynamic changes suggest that S100a4 may contribute to orchestrating HFSC the transition from quiescence to an active state while also facilitating their differentiation during regeneration.

Previous studies have demonstrated that S100A4 interacts with the cytoskeletal components such as non-muscle myosin heavy chain (NMMHC) IIA, tropomyosin, and actin [23,36], thereby influencing cytoskeletal organization, cell morphology, and migration behavior. Furthermore, S100A4 has been implicated in the phenotypic transitions among keratocytes, fibroblasts, and myofibroblasts during corneal wound healing [37], suggesting a role in modulating cytoskeletal and epithelial–mesenchymal transition (EMT)-associated signaling pathways. Other S100 family members, including S100A8/A9, have been shown to interact with keratins in epithelial cells, mediating Ca^2+^-dependent reorganization of cytoskeletal filaments, which is critical for differentiation and inflammatory activation [26]. Keratins such as KRT5 and KRT18 have been implicated in controlling stem cell adhesion, polarity, and lineage commitment through their roles in cytoskeletal organization and signal transduction including Wnt/β-catenin and MAPK [38,39]. Mechanistically, in the present study, S100A4 was found to interacts directly with key keratins, including KRT5, KRT14, KRT8, and KRT18—proteins essential for epithelial integrity and hair follicle development [40,41,42,43]. This interaction indicates that S100A4 may influence HFSC behavior not only by modulating keratin expression and organization, but also by affecting keratin-associated signaling pathways that guide stem cell fate decisions.

Our findings indicate that S100a4 influences both stem cell activation and differentiation. The transient upregulation of S100a4 following skin injury and hair regrowth suggests a role in regeneration rather than steady-state maintenance. In this context, S100a4 may function to modulate HFSC states by promoting cellular plasticity and transitions between multipotent and lineage-committed states. Similar to its reported roles in phenotypic transitions [37], S100a4 may help maintain cells in a dynamically unstable or transitional condition conducive to tissue remodeling. Future studies, such as single-cell analyses or co-immunofluorescence will be valuable to clarify how S100a4 regulate these processes.

Together, our study identifies S100A4 as a modulator of HFSC state transitions, influencing cytoskeletal architecture and signaling to regulate stem cell activation and differentiation. These findings reveal a previously unrecognized function for S100a4 in secondary HFSC biology and highlight its potential as a target for enhancing cashmere fiber production.

## 4. Materials and Methods

### 4.1. Experimental Animals

All experimental cashmere goats were from the Department of Agricultural and Rural Affairs of Liaoning Province, Shenyang. Six cashmere goats (three male and three female) that were 1.5 years old were selected in October.

### 4.2. Cell Isolation and Culture

Secondary HFSCs were isolated as previously described [44]. Secondary HFSCs and hair matrix cells were cultured in DMEM/F12 (Gibco, Grand Island, NY, USA), 10% Foetal Bovine Serum (Mylscience Biotechnology Co., Ltd., Shenyang, China), 10 ng/mL EGF, 10 ng/mL insulin, and 0.4 µg/mL hydrocortisone.

### 4.3. Immunofluorescence Assay of Secondary HFSCs

Secondary HFSCs at passage 2 were seeded into 6-well plates and cultured for 24 h. Cells were then fixed with 4% paraformaldehyde for 10 min at room temperature, permeabilized with 0.5% Triton X-100 for 20 min, and blocked with 1% BSA (Boster, Pleasanton, CA, USA) at room temperature for 1 h. After blocking, cells were incubated overnight at 4 °C with an anti-KRT15 primary antibody (1:250, Proteintech, Rosemont, IL, USA). After antibody incubation, samples were washed three times with PBS for 5 min each at room temperature. Following washing, cells were incubated with Goat Anti-Rabbit IgG H&L (Alexa Fluor 488) (1:1000, Abcam, Cambridge, UK) for 2 h at room temperature. Nuclei were counterstained with DAPI (Sangon Biotech, Shanghai, China) for 10 min, and images were acquired using a fluorescence microscope.

### 4.4. Cell Culture and Treatment

To knockdown or increase *S100a4*, *S100a4*-specific shRNA was cloned into vector PLVX or *S100a4* was inserted into the MSCV vector. And then lentiviral transfection was conducted to achieve *S100a4* overexpression (OE*S100a4*) or *S100a4* knockdown (sh*S100a4*), scramble sequence to achieve overexpression control (OECtrl) or *S100a4* knockdown control (shCtrl). The primers used are shown in Table S1. Lentiviral construct pSPAX2 and pMD2G were co-transfected into HEK293T cells using LipoFiter (Hanbio, Shanghai, China) to obtain lentiviral particles. The secondary HFSCs were infected with OE*S100a4* or sh*S100a4* lentiviral, stable clones of knockdown cells were selected with puromycin (Hanbio, Shanghai, China).

### 4.5. Quantitative Real-Time PCR

Total RNA was extracted from secondary HFSCs using RNAiso (Takara, Beijing, China). cDNA was synthesized from the extracted RNA using PrimeScript™ RT (Takara, Dalian, China), following the manufacturer’s recommended protocol. Quantitative real-time PCR (RT-qPCR) was performed with SYBR Premix Ex Taq II (Takara, Dalian, China). Relative mRNA expression levels of target genes were calculated using the 2^−ΔΔCt^ method, with *Gapdh* serving as the internal reference control. Statistical analyses were conducted using SPSS software (v.15.0). Data are presented as mean ± SD, and statistical significance was set at *p* < 0.05. The detailed sequences of the primers used in this study are provided in Table S2.

### 4.6. GST Pull-Down Assay and LC-MS/MS Analysis

Protein extracts for GST pull-down assays were isolated from secondary HFSCs and HEK293T cells using a cell protein extraction kit (Epizyme, Shanghai, China). For exogenous protein expression, HEK293T cells were transfected with pCMV-N-HA-KRT5, pCMV-N-HA-KRT14, pCMV-N-HA-KRT8, or pCMV-N-HA-KRT18 plasmids. Subsequently, GST-tag resin alone (NC), purified GST (GST), and GST-S100A4 were incubated under identical conditions with HA-KRT5, KRT14, KRT8, and KRT18 fusion proteins overnight at 4 °C. After overnight incubation at 4 °C, beads were washed three times for 5 min each with binding buffer (50 mM Tris-HCl, 200 mM NaCl, 10 mM MgCl_2_, 0.1% NP-40, 1 mM DTT, 1 mM EDTA), centrifuged at 4 °C, 1000× *g* for 10 s between washes, and then eluted with elution buffer and Laemmli loading buffer (Sigma-Aldrich, Sydney, NSW, Australia). Finally, the eluted protein samples were analyzed by LC–MS/MS and immunoblotting for protein identification and validation. All the primers used for GST pull-down are listed in Table S3.

### 4.7. Statistical Analysis

The *t*-test was employed to analyze the significance of RT-qPCR data, and GraphPad Prism software (v.8.0.1) was used in the dual-luciferase reporter assay. A *p* value of <0.05 was considered statistically significant.

## 5. Conclusions

In summary, this study identifies S100a4 as a central regulator of secondary HFSC activity in cashmere goats. We show that S100a4 is highly enriched in secondary HFSCs and exhibits dynamic, stage-specific expression throughout the hair cycle, underscoring its importance in coordinating secondary hair follicle regeneration. Functionally, S100A4 functions as a modulator of HFSC state transitions, influencing cytoskeletal architecture and signaling to regulate stem cell activation and differentiation. These findings advance our understanding of the molecular mechanisms governing hair follicle cycling and highlight S100a4 as a promising target for strategies aimed at improving cashmere fiber production.

## Data Availability

The original contributions presented in this study are included in the article/Supplementary Materials. Further inquiries can be directed to the corresponding author.

## Supplementary Materials

The following supporting information can be downloaded at: https://www.mdpi.com/article/10.3390/ijms27020849/s1.
