# Supplementary material for: Molecular Regulation of Secondary Hair Follicle Stem Cell by S100a4 in Cashmere Goat
Source: Int J Mol Sci. 2026 Jan 15;27(2):849. doi: 10.3390/ijms27020849 (PMC12841439; doi:10.3390/ijms27020849)
Supplement: Supplementary file 1 [file ijms-27-00849-s001.zip › ijms-4067335-supplementary.pdf]

Table S1. Primer sequences for S100a4 knockdown, overexpression, and corresponding controls

Name	Primer sequence (5′ to 3′)
sh_*S100a4*-F	GATCCGGCTTCGAAGGGTTCCCTGATAACTCGAGTTATCAGGGAACCCTTCGAAGTTTTT
sh_*S100a4*-R	AATTAAAAACTTCGAAGGGTTCCCTGATAACTCGAGTTATCAGGGAACCCTTCGAAGCCG
sh_*Ctrl*-F	GATCCGGTTCTCCGAACGTGTCACGTAACTCGAGTTACGTGACACGTTCGGAGAATTTTT
sh_*Ctrl*-R	AATTAAAAATTCTCCGAACGTGTCACGTAACTCGAGTTACGTGACACGTTCGGAGAACCG
OE_*S100a4*-F	CCGGAATTCATGGCATACCCCCTGGAGA
OE_*S100a4*-R	CGCGGATCCTCATTTTTTCCGGGGCTG
OE_Ctrl-F	GCAGTTTCTAGCGAACCATC
OE_Ctrl-R	CGCTCTGCCCACTGACGG

Table S2. Primer sequences for mRNA RT-qPCR

Name	Forward primer sequence (5′ to 3′)	Reverse primer sequence (5′ to 3′)
S100a4	GCAAGGAGGGTGACAAG	GGACAGGAAGACGCAGTA
Klk5	TTGTGAATGGGACCGACTG	GGATTTGATGCCCTGGAA
Cux1	GACCCAGATGTCCACCAC	TCGGCCTTGGCAGTAA
Ivl	GCGGAGCCTCAGAAAGT	TGGACCTGGTTGGGAGC
Itga6	TCCATTACATTGCGTTTG	TCCACTATGATTGGCTCT
Krt15	GCTTTGGTGGGGGTTTTGGC	CAAGCGGTCATTGAGATTC
Krt14	GCGTGGGTAGTGGTTTTGGT	CCGTAAGAGCGGAAACA
Ki67	AAGAGGCAGCCCAAGTCATC	TTCTTAGCCTACTCCGGCCT
Pcna	AAGCCACTCCACTGTCTCCT	CATCCTCGATCTTGGGAGCC
Cdk1	TTCTATCCCTCCTGGTC	TTGGGAAATGTACTCTTGT
Gapdh	TTGTGATGGGCGTGAACC	CCCTCCACGATGCCAAA

Table S3. Primer sequences for vector construction

Name	Forward primer sequence (5′ to 3′)	Reverse primer sequence (5′ to 3′)
S100a4	CGGGATCCATGGCATACCCCCTGGAGA	CCCAAGCTTTCATTTTTTCCGGGGCTGCTTA
Krt5	CGCGGATCCATGTCTCGCCAGT	CCGGAATTCTTAGCTCTTGAA
Krt14	CGCGGATCCATGCCAGGCCCGC	CCGGAATTCTTAGTTCTTGGTGCGAACG
Krt8	CGCGGATCCATGTCCATCAGGGT	CCGGAATTCTCACTTGGACAGGA
Krt18	CGCGGATCCATGAGCTTCAGCGCCC	CCGGAATTCTCAGTGCCTCAGAACTTTGG
